# Avian Metapneumovirus Subgroup C Phosphoprotein Suppresses Type I Interferon Production by Blocking Interferon Regulatory Factor 3 Nuclear Translocation

**DOI:** 10.1128/spectrum.03413-22

**Published:** 2022-12-20

**Authors:** Lei Hou, Yongyan Shi, Jinshuo Guo, Tong Sun, Dedong Wang, Xiaoyu Yang, Changzhe Liu, Yongqiu Cui, Ning Zhu, Xinxin Tong, Yang Pan, Xufei Feng, Jianwei Zhou, Jue Liu

**Affiliations:** a College of Veterinary Medicine, Yangzhou University, Yangzhou, China; b Jiangsu Co-Innovation Center for Prevention and Control of Important Animal Infectious Diseases and Zoonoses, Yangzhou University, Yangzhou, China; c College of Animal Science and Technology, Anhui Agricultural University, Hefei, China; University of Siena

**Keywords:** phosphoprotein, IFN-β production, IRF3, nuclear translocation, avian metapneumovirus subgroup C

## Abstract

Avian metapneumovirus subgroup C (aMPV/C) is an important pathogen that causes upper respiratory symptoms and egg production decline in turkeys and chickens. aMPV/C infection leads to inhibition of the host antiviral immune response. However, our understanding of the molecular mechanisms underlying host immune response antagonized by aMPV/C infection is limited. In this study, we demonstrated that the aMPV/C phosphoprotein (P) inhibits the IFN antiviral signaling pathway triggered by melanoma differentiation gene 5 (MDA5) and reduces interferon β (IFN-β) production and IFN-stimulated genes (ISGs) by targeting IFN regulatory factor 7 (IRF7) but not nuclear factor κB (NF-κB) in DF-1 cells. Moreover, we found that aMPV/C P protein only blocks the nuclear translocation of IRF3 by interacting with IRF3 in HEK-293T cells, instead of affecting IRF3 phosphorylation and inducing IRF3 degradation, which suppresses IRF3 signaling activation and results in a decrease in IFN-β production. Collectively, these results reveal a novel mechanism by which aMPV/C infection disrupts IFN-β production in the host.

**IMPORTANCE** The innate immune response is the first defense line of host cells and organisms against viral infections. When RNA viruses infect cells, viral RNA induces activation of retinoic acid-induced gene I and melanoma differentiation gene 5, which initiates downstream molecules and finally produces type I interferon (IFN-I) to regulate antiviral immune responses. The mechanism for avian metapneumovirus (aMPV) modulating IFN-I production to benefit its replication remains unknown. Here, we demonstrate that phosphoprotein of aMPV subgroup C (aMPV/C) selectively inhibits the nuclear translocation of interferon regulatory 3 (IRF3), instead of affecting the expression and phosphorylation of IRF3, which finally downregulates IFN-I production. This study showed a novel mechanism for aMPV/C infection antagonizing the host IFN response.

## INTRODUCTION

Avian metapneumovirus (aMPV) is an emerging pathogen that causes respiratory tract symptoms and egg production decline in both turkeys and chickens (1). aMPV was originally identified in South Africa (2), following which it spread worldwide, resulting in huge economic losses to the global poultry industry (3, 4). aMPV, belonging to the family Paramyxoviridae, has a single-stranded, negative-sense RNA, nonsegmented genome (5), which encodes eight proteins (N, P, M, F, M2, SH, G, and L protein, respectively) (6). According to sequence analysis and antigenic divergence among the aMPV strains, aMPV isolates were divided into four subgroups (A, B, C, and D) (7). Interestingly, the genetic evolutionary relationship between aMPV/C and human metapneumovirus (hMPV) is closer than that between other subgroups (8 to 11). aMPV/C infections have been reported in the United States (12) and subsequently in South Korea (13). The JC strain of aMPV/C, a causative agent for severe respiratory symptoms, has been isolated from broilers in southern China (11), and this strain can infect mice and activate strong inflammation in the lungs (14). Additionally, aMPV/C infection degrades the mitochondrial antiviral signaling (MAVS) protein via ubiquitination (15).

The innate immune response is the first line of defense of host cells and organisms against viral infections, in which IFN or IFN-mediated antiviral responses play a key role (16). The pathogen-associated molecular pattern (PAMP) is mainly double-stranded RNA (dsRNA) during the RNA virus infection. When RNA viruses infect cells, the host pattern recognition receptors recognize these PAMPs and initiate a cascade of innate immune responses (17). For example, retinoic acid-induced gene I (RIG-I) and melanoma differentiation gene 5 (MDA5) are induced by recognized viral RNA, which activates downstream molecules MAVS, TANK binding kinase 1 (TBK1), I-kappa B kinase ε (IKKε), nuclear factor κB (NF-κB), and interferon regulatory factor 3 (IRF3), ultimately inducing IFN-I to regulate antiviral immune responses (18 to 21).

Viruses have developed many mechanisms to avoid or resist the innate immune response of the host and break through the first line of host defense. Viral proteins play an important role in this process. For example, Seneca valley virus (SVV) has been shown to decrease IFN production by 3C-protease-mediated cleavage of MAVS, TRIF, and TANK (22). Porcine bocavirus (PBoV) NP1 protein has been found to interfere with IRF3 DNA-binding activity (23). Moreover, many viruses of the family Paramyxoviridae have been reported to subvert host defense. The N and P proteins of peste des petits ruminants virus (PPRV) inhibit IFN-β production by blocking IRF3 activation and dysregulating the RIG-I pathway, respectively (24, 25). The measles virus (MV) P and V proteins impede phosphorylation of the signal transducer and activator of transcription 1 (STAT1) by Janus kinase 1 (JAK1), affecting the IFN response (26). Additionally, the Nipah virus (NiV) P protein and Newcastle disease virus (NDV) V protein inactivate STAT1 in the nucleus and degrade MAVS by ubiquitination, respectively (27, 28). The P protein, which blocks RIG-I-mediated sensing of viral RNA, is responsible for the failure of the IFN-I response in hMPV-B1 infection (29). The above inhibitory roles of the P protein and the close evolutionary relationship between aMPV and hMPV prompted us to explore the mechanism by which the P protein suppresses IFN production.

In this study, the effect of the aMPV/C P protein on the inhibition of the IFN response was investigated. We found that IFN production and interferon-stimulated gene (ISG) expression were inhibited by aMPV/C P protein, and that IRF3 was identified as the target protein of P protein. We further determined that the aMPV/C P protein inhibited the translocation of IRF3 from the cytoplasm into the nucleus by interacting with IRF3. Collectively, these results reveal a novel mechanistic link between the aMPV/C P protein and IRF3 nuclear translocation, which provides a prevention and control strategy for aMPV infection.

## RESULTS

### aMPV/C P protein suppresses IFN-I production.

**FIG 1:**
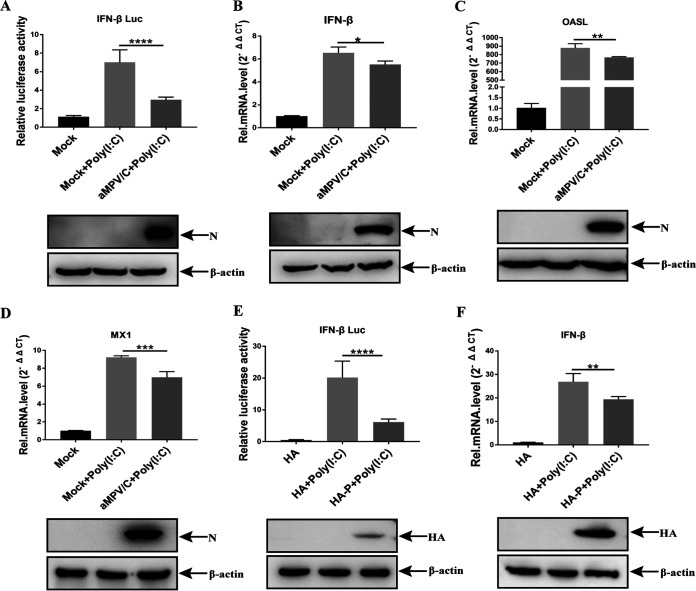
aMPV/C P protein suppresses IFN-β production. (A) DF-1 cells cotransfected with IFN-β-luc and pRL-TK for 6 h were infected with aMPV/C. At 18 h postinfection (hpi), the cells were transfected with poly (I·C) for 12 h; subsequently, they were processed, and the luciferase activity was measured using a dual-luciferase assay. The expressions of viral N protein and β-actin were detected by Western blotting. (B to D) DF-1 cells were mock infected or infected with aMPV/C for 30 h; subsequently, the expression of IFN-β, OASL, and MX1 mRNA were measured by RT-qPCR. The expression of HA-tag protein and β-actin were detected by Western blotting. (E) DF-1 cells cotransfected with IFN-β-luc and pRL-TK, together with expression plasmid P (HA-P), or HA-vector plasmid (HA). At 24 h transfection (hpt), the cells were transfected with poly (I·C) for 12 h, following which the luciferase activity was measured and the expressions of HA-tag protein and β-actin were detected by Western blotting. (F) DF-1 cells were transfected with expression plasmid P (HA-P) or empty plasmid (HA). At 24 hpt, the cells were transfected with poly (I·C) for 12 h, following which the expression of IFN-β mRNA was measured by RT-qPCR. The expressions of HA-tag protein and β-actin were detected by Western blotting. Values are presented as the mean ± SD from three independent experiments. Statistical analysis was performed using Student's *t* test (*, *P < *0.05; **, *P < *0.01; ***, *P < *0.001; ****, *P < *0.0001).

IFN-I is responsible for the host antiviral response *in vivo* and *in vitro* through the mediation of a set of ISGs that inhibit viral replication (30, 31). To investigate the relationship between aMPV/C infection and IFN-I production, IFN-β reporter activity and IFN-β mRNA levels were analyzed by luciferase reporter assay and RT-qPCR in aMPV/C-infected or mock-infected DF-1 cells. Cells treated with high-molecular-weight poly (I·C), an IFN inducer, served as a positive control for IFN production. aMPV/C infection significantly decreased the activity of the IFN-β promoter and the level of IFN-β mRNA in the presence of poly (I·C) stimulation, and the expression of N protein represented aMPV/C infection (Fig. 1A to B), indicating that aMPV/C infection suppressed the IFN-β response. RIG-I is activated after binding dsRNA, mediating downstream IFN-β production, and finally initiating a series of IFN-stimulated genes (ISGs) (32). However, the lack of RIG-I protein in chicken cells indicates that MDA5 acts as a complementary ligand in the recognition of RNA (33). 2′-5′-oligoadenylate synthetase-like (OASL) and myxovirus resistance 1 (MX1) are two of the most induced proteins in cells following IFN-I or viral infection (34). To further confirm the inhibitory effect of aMPV/C infection on IFN-I production, the mRNA levels of OASL and MX1 were measured using RT-qPCR. The results are consistent with the downregulation of IFN-β induced by the virus; aMPV/C infection significantly reduced poly (I·C)-induced OASL and MX1 mRNA (Fig. 1C to D).

The phosphoprotein (P) protein of the family *Paramyxoviridae* has been reported to perform an inhibitory role in the IFN signaling pathway, including the P proteins of PPRV (25), MV (26), NiV (27), and hMPV (29). Moreover, the genetic evolutionary relationship between hMPV and aMPV/C was very close (9, 11), suggesting that the aMPV/C P protein may also exhibit a similar inhibitory effect. Thus, this suggestion prompted us to explore the effect of the aMPV/C P protein on IFN production. DF-1 cells were transfected with HA-P expression plasmids or HA-vector plasmids and subsequently transfected with poly (I·C) to analyze IFN-β levels. As shown in Fig. 1E to F, the poly (I·C)-mediated increase in IFN-β promoter activity and IFN-β mRNA was inhibited by the expression of P protein. These results indicate that the aMPV/C P protein plays an important role in the inhibition of IFN production by aMPV/C infection.

### aMPV P protein targets IRF7 for inhibiting IFN-β activation.

**FIG 2:**
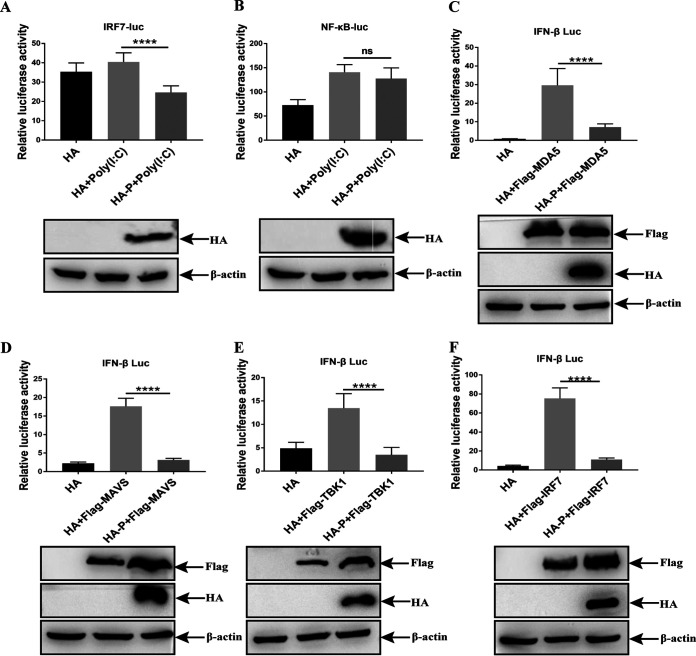
aMPV P protein targets IRF7 for inhibiting IFN-β activation in DF-1 cells. (A and B) DF-1 cells were cotransfected with IRF7-luc or NF-κB-luc and pRL-TK, expression plasmid P (HA-P), or empty plasmid (HA). At 24 hpt, the cells were transfected with poly (I·C) for 12 h and subsequently subjected to luciferase activity measurement. The expressions of HA-tag protein and β-actin were detected by Western blotting. (C to F) DF-1 cells were cotransfected with MDA5, MAVS, TBK1, or IRF7 expression plasmids or empty plasmid (HA), IFN-β-luc, and pRL-TK. At 36 hpt, the cells were processed and the luciferase activity measured. The expression of HA-tag protein and Flag-tag protein were detected by Western blotting. Values are presented as the mean ± SD from three independent experiments. Statistical analysis was performed using Student's *t* test (ns, *P* > 0.05; ****, *P < *0.0001).

Activation of IRF7 and NF-κB regulates IFN-β production in chicken cells (35). To clarify the inhibitory mechanism of IFN-β production by the aMPV/C P protein, DF-1 cells were cotransfected with IRF7-luc or NF-κB-luc and pRL-TK, together with expression plasmid P (HA-P), or empty plasmid (HA), and subsequently treated with poly (I·C). The promoter activities of IRF7 and NF-κB were measured using a luciferase reporter assay. The results showed that the P protein downregulated poly (I·C)-mediated increase in IRF7 luciferase activity but did not change the expression of the NF-κB-dependent reporter gene (Fig. 2A to B), indicating that the aMPV/C P protein suppresses the IRF7 pathway but not the NF-κB pathway.

In DF-1 cells, signaling components such as MDA5, MAVS, TBK1, and IRF7 are involved in regulating IRF7 signaling activation. To screen the molecule inhibited by P protein, we cotransfected the cells with the expression plasmids of Flag-MDA5, Flag-MAVS, Flag-TBK1, or Flag-IRF7 and HA-P, together with the IFN-β-dependent reporter gene. Expression of all proteins obviously induced IFN-β promoter activity. However, IFN-β luciferase activity was downregulated in cells expressing the P protein (Fig. 2C to F). Importantly, the expression of the P protein decreased IFN-β activation mediated by IRF7. Therefore, we speculated that aMPV/C P protein suppressed IFN-β production by targeting IRF7.

### aMPV/C P protein interacts with the AD domain (143 to 303 aa [amino acids]) of IRF7.

**FIG 3:**
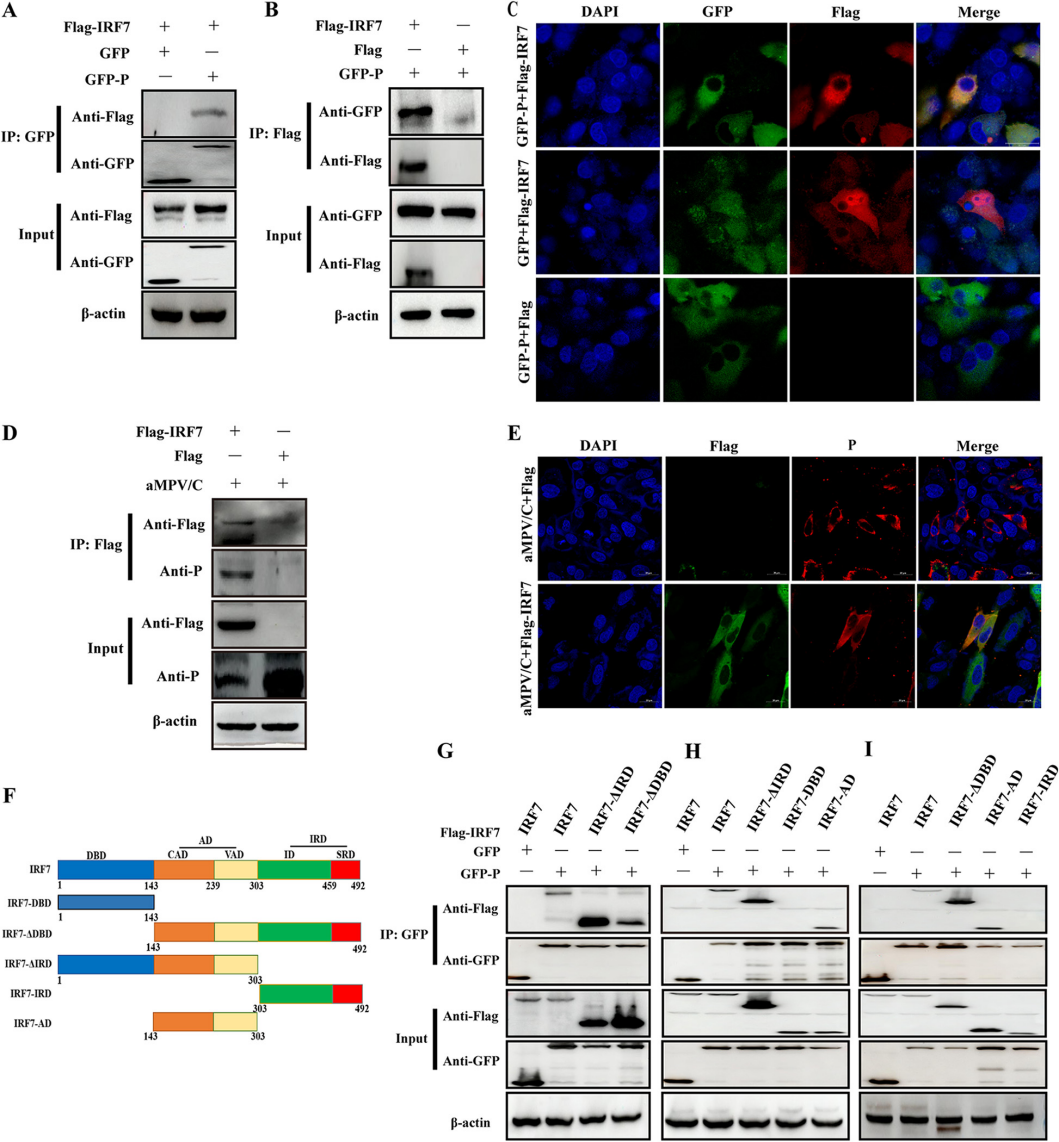
aMPV P protein interacted with IRF7. (A and B) HEK-293T cells were cotransfected with GFP-P and Flag-IRF7 expression plasmids for 36 h. The cells were processed, immunoprecipitated with anti-GFP, anti-Flag, or anti-β-actin antibody, and subsequently analyzed using Western blotting with anti-Flag, anti-GFP, or anti-β-actin antibody. (C) DF-1 cells were cotransfected with GFP-P and/or Flag-IRF7 expression plasmids for 36 h, fixed, and processed. Dual labeling for GFP-P (green) and Flag-IRF7 (red) were observed by immunostaining with anti-Flag antibody. Cell nuclei were stained with DAPI (blue). The yellow observed in the merged images was considered to indicate colocalization areas. Scale bars, 20 μm. (D) DF-1 cells were transfected with Flag-IRF7 or Flag expression plasmids for 12 h, followed by infection with aMPV/C for 72 h. The cells were processed, immunoprecipitated with anti-Flag antibody, and subsequently analyzed using Western blotting with anti-Flag or anti-P antibody. (E) DF-1 cells were treated with Flag-IRF7 or Flag plasmids and aMPV/C as described in Fig. 3D, fixed, and processed. Dual labeling for P (red) and Flag- (green) were observed by immunostaining with anti-P or anti-Flag antibody. Cell nuclei were stained with DAPI (blue). The yellow observed in the merged images indicates colocalization areas. Scale bars, 20 μm. (F) Schematic representation of chicken IRF7 at different lengths, including full-length IRF7 (aa 1 to 492), IRF7 DBD (aa 1 to 143), IRF7 ΔDBD (aa 143 to 492), IRF7 ΔIRD (aa 1 to 303), IRF7 IRD (aa 303 to 492), and IRF7 AD (CAD+VAD) (aa 143 to 303). (G) HEK-293T cells were cotransfected with GFP-P and Flag-IRF7, IRF7 ΔDBD, or IRF7 ΔIRD expression plasmids for 36 h. The cells were processed, immunoprecipitated with anti-GFP or anti-Flag antibody, and analyzed using Western blotting with anti-Flag, anti-GFP, or anti-β-actin antibody. (H) HEK-293T cells were cotransfected with GFP-P and Flag-IRF7, IRF7 ΔIRD, IRF7 DBD, or IRF7 AD expression plasmids for 36 h. The cells were processed, immunoprecipitated with anti-GFP, anti-Flag, or anti-β-actin antibody, and analyzed using Western blotting with anti-Flag or anti-GFP antibody. (I) HEK-293T cells were cotransfected with GFP-P and Flag-IRF7, IRF7 ΔDBD, IRF7 AD, or IRF7 IRD expression plasmids for 36 h. The cells were processed, immunoprecipitated with anti-GFP or anti-Flag antibody, and analyzed using Western blotting with anti-Flag, anti-GFP, or anti-β-actin antibody.

The inhibition of IRF7 activity by P protein prompted us to investigate the possibility of an interaction between P and IRF7. HEK-293T cells were cotransfected with expression plasmids GFP-P and Flag-IRF7 or GFP and Flag-IRF7, and a coimmunoprecipitation (co-IP) assay was performed with anti-GFP and anti-Flag antibodies. A specific interaction was observed between GFP-P and Flag-IRF7, whereas no band was observed between GFP and Flag-IRF7 (Fig. 3A). A reverse co-IP experiment was performed with anti-FLAG and anti-GFP antibodies; the interaction between these two proteins was determined (Fig. 3B). Confocal immunofluorescence microscopy showed that GFP-P colocalized with Flag-IRF3 in the cytoplasm (Fig. 3C). To further confirm this interaction between P protein and IRF7, DF-1 cells transfected with Flag-IRF7 or Flag plasmids were infected with aMPV/C, and a co-IP assay was performed with anti-Flag antibodies. The results showed that Flag-IRF7 coprecipitated with aMPV/C P protein in virus-infected DF-1 cells, whereas no bands appeared in Flag-expressing cells (Fig. 3D). Moreover, the results of confocal immunofluorescence microscopy showed that P and Flag-IRF7 colocalized in the cytoplasm, which is similar to the subcellular locations of GFP-P and Flag-IRF7 (Fig. 3E). These results strongly confirmed the interaction between the aMPV/C P protein and host IRF7 protein.

From the N-terminal to C-terminal, IRF7 successively contains multiple domains, such as DNA-binding domain (DBD), constitutive activation domain (CAD), virus-activated domain (VAD), inhibitory domain (ID), and signal response domain (SRD) (36, 37). To further identify the key domains of IRF7 that interact with the P protein, a series of truncated plasmids of different domains were constructed (Fig. 3F). We first analyzed the possibility of interaction between the P protein and IRF7 ΔID+SRD (1 to 303 aa) or IRF7ΔDBD (143 to 492 aa) by cotransfecting GFP-P or GFP with full-length Flag-IRF7 (1 to 492 aa), IRF7 ΔID+SRD, and IRF7ΔDBD, followed by co-IP. The results showed that both IRF7 ΔID+SRD and IRF7 ΔDBD interacted with the P protein (Fig. 3G), indicating that the AD region (CAD+VAD) of IRF7 may be a critical interaction domain of the P protein. On the basis of this hypothesis, we subdivided IRF7 into IRF7 DBD (1 to 143 aa), IRF7 AD (143 to 303 aa), and IRF7 IRD (303 to 492 aa) (Fig. 3F). In cells expressing proteins with the different domains, only the IRF7 AD (143 to 303 aa) can interact with the P protein, indicating that the AD (143 to 303 aa) domain of IRF7 is key for the inhibitory function of the P protein (Fig. 3H and I).

### aMPV P protein interacts with IRF3 and targets IRF3 for inhibiting IFN-β activation in HEK-293T cells.

**FIG 4:**
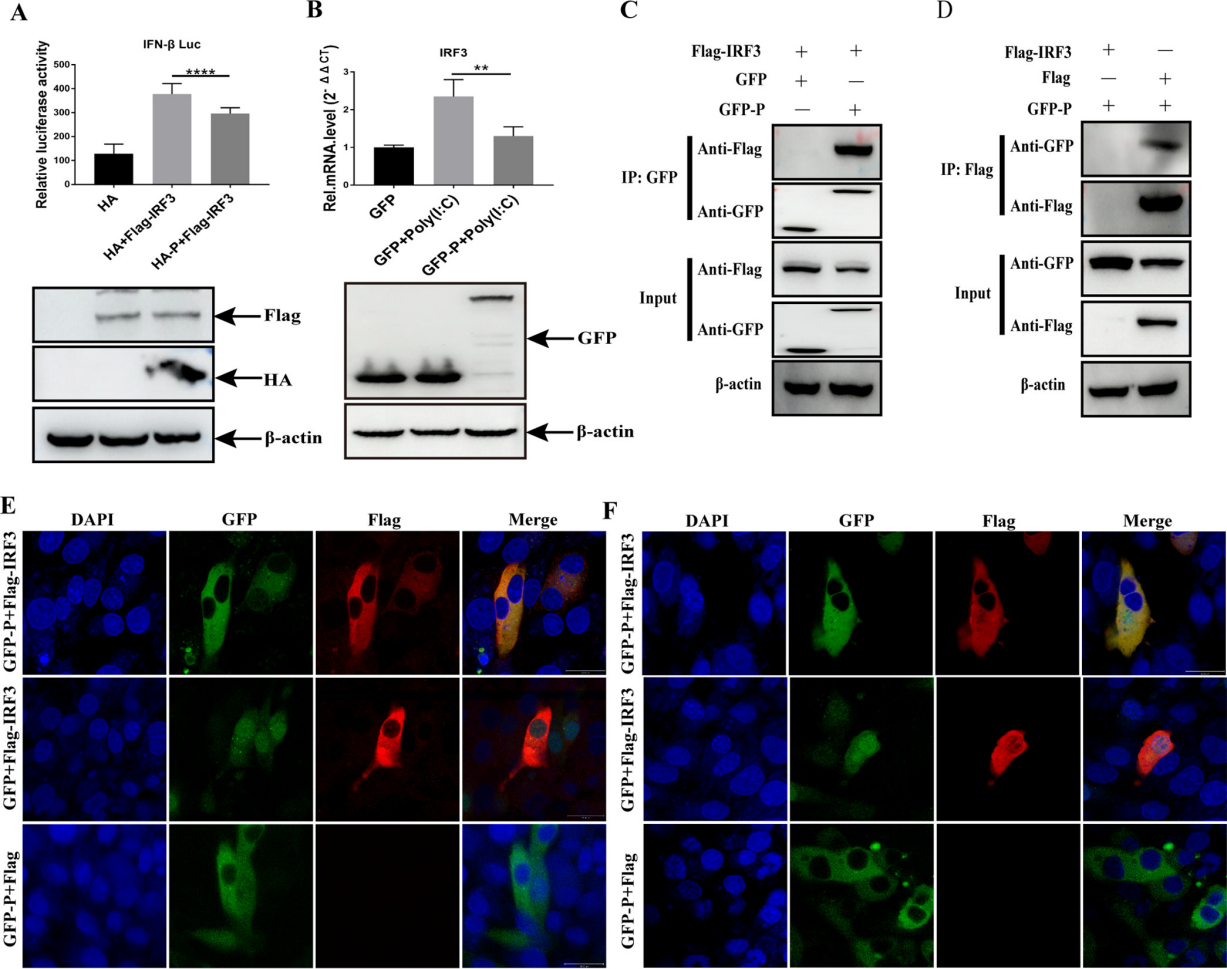
aMPV P protein targets IRF3 for inhibiting IFN-β activation in HEK-293T cells. (A) HEK-293T cells cotransfected with IRF3-luc, pRL-TK, expression plasmid P (HA-P), or empty plasmid (HA). At 24 hpt, the cells were transfected with poly (I·C) for 12 h and the luciferase activity measured; the expression of HA-tag protein and β-actin were detected using Western blotting. (B) HEK-293T cells were transfected with expression plasmid P (GFP-P) or empty plasmid (GFP). At 24 hpt, the cells were transfected with poly (I·C) for 12 h, and the expression of IRF3 mRNA was measured using RT-qPCR. (C and D) HEK-293T cells were cotransfected with GFP-P and Flag-IRF3 expression plasmids for 36 h. The cells were processed, immunoprecipitated with anti-GFP, anti-Flag, or anti-β-actin antibody, and analyzed using Western blotting with anti-Flag, anti-GFP, or anti-β-actin antibody. (C and D) HEK-293T cells were cotransfected with GFP-P and Flag-IRF3 expression plasmids for 36 h. The cells were processed, immunoprecipitated with anti-GFP or anti-Flag antibody, and subsequently analyzed using Western blotting with anti-Flag or anti-GFP antibody. (E and F) HEK-293 or A549 cells were cotransfected with GFP-P and/or Flag-IRF3 expression plasmids for 36 h, fixed, and processed. The dual labeling for GFP-P (green) and Flag-IRF3 (red) was observed using immunostaining with anti-Flag antibody. Cell nuclei were stained with DAPI (blue). The yellow observed in the merged images is considered to indicate colocalization areas. Scale bars, 20 μm. Values are presented as the mean ± SD from three independent experiments. Statistical analysis was performed using Student's *t* test (**, *P < *0.01; ****, *P < *0.0001).

Although IRF3 is absent in chickens and other avian species (38 to 40), the role of mammal-derived IRF3 in IFN induction is similar to that of IRF7 in chickens and other avian species (41). Therefore, we explored whether aMPV/C P protein can block IRF3 activation to inhibit IFN production in mammalian cells (HEK-293T cells). First, the effect of the P protein on IFN-β promoter activity mediated by IRF3 was evaluated. HEK-293T cells were cotransfected with HA-P- or HA-vector plasmids and Flag-IRF3 along with the IFN-β luciferase reporter system. Overexpression of IRF3 significantly enhanced IFN-β promoter activity, whereas the P protein suppressed this activation (Fig. 4A). To further verify the inhibitory role of the P protein in IRF3 activity, IRF3 mRNA levels were measured by RT-qPCR. The results showed that IRF3 mRNA was strongly downregulated in the P-expressing cells (Fig. 4B). Overall, consistent with the inhibition of IRF7 activity by P protein in DF-1 cells, IRF3 activation was inhibited by the P protein in HEK-293T cells.

The interaction between the aMPV/C P protein and IRF7 prompted us to investigate whether the P protein interacts with IRF3. HEK-293T cells were cotransfected with GFP-P or GFP-vector plasmids and Flag-IRF3 plasmids, followed by co-IP assay with an anti-GFP antibody. P protein coprecipitated with IRF3, whereas no bands appeared in GFP-expressing cells (Fig. 4C). Reverse co-IP assay further confirmed these results (Fig. 4D). To further determine whether the subcellular locations of the P protein and IRF3 were similar to those of the P protein and IRF7, HEK-293T or A549 cells were cotransfected with GFP-P and Flag-IRF3, or GFP and Flag-IRF3; the results showed that GFP-P and Flag-IRF3 colocalized in the cytoplasm (Fig. 4E and F). Collectively, these results suggest that the inhibitory effect of the P protein against IFN-β production is the same regardless of whether it is mediated by IRF7 or IRF3.

### aMPV P protein (101 to 200 aa) interacts with IRF3.

**FIG 5:**
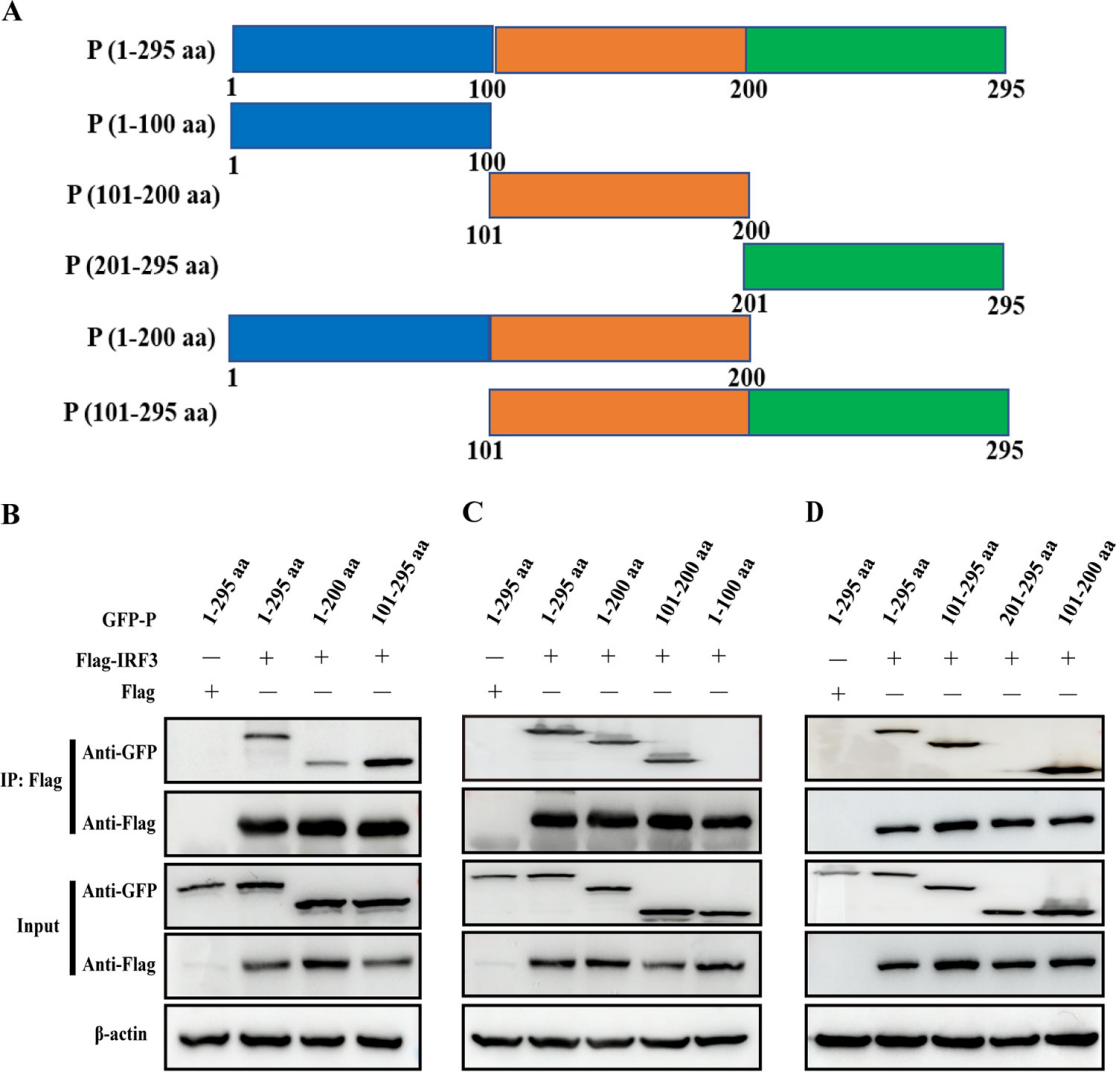
aMPV/C P protein (101 to 200 aa) interacts with IRF3. (A) Schematic representation of aMPV/C P protein (aa 1 to 295) and various truncated P proteins (aa 1 to 200, aa 101 to 295, aa 1 to 100, aa 101 to 200, and aa 201 to 295). (B) HEK-293T cells were cotransfected with GFP-P, GFP-P (1 to 200 aa), or GFP-P (101 to 295 aa) and Flag-IRF3 plasmids for 36 h. The cells were processed, immunoprecipitated with anti-Flag antibody, and analyzed using Western blotting with anti-Flag, anti-GFP antibody, or anti-β-actin. (C) HEK-293T cells were cotransfected with GFP-P, GFP-P (1 to 100 aa), GFP-P (101 to 200 aa), or GFP-P (1 to 200 aa) and Flag-IRF3 plasmids for 36 h. The cells were processed, immunoprecipitated with anti-Flag antibody, and analyzed using Western blotting with anti-Flag, anti-GFP, or anti-β-actin antibody. (D) HEK-293T cells were cotransfected with GFP-P, GFP-P (101 to 200 aa), GFP-P (201 to 295 aa), or GFP-P (101 to 295 aa) and Flag-IRF3 plasmids for 36 h. The cells were processed, immunoprecipitated with anti-Flag antibody, and analyzed using Western blotting with anti-Flag, anti-GFP, or anti-β-actin antibody.

Based on the results of interaction between P protein and IRF3, the critical interaction domain in P protein was further analyzed. To identify the key domains of P protein, it was randomly truncated into the different domains (1 to 200, 101 to 295, 1 to 100, 101 to 200, and 201 to 295) (Fig. 5A), and plasmids corresponding to different truncated domains were successfully constructed. HEK-293T cells were cotransfected with expression plasmids Flag-IRF3 or Flag and the full-length P protein or truncated P protein (1 to 200 and 101 to 295 aa), and the co-IP assay was analyzed with anti-GFP and anti-Flag antibodies. As shown in Fig. 5B, both P protein (1 to 200 aa) and P protein (101 to 295 aa) interacted with IRF3, indicating that the P protein (101 to 200 aa) may be a key domain interacting with IRF3. To confirm this hypothesis, the subdivided P protein truncates (1 to 100, 101 to 200, and 201 to 295) were analyzed. In cells expressing P protein truncates, the specific interaction only appeared at the position of amino acids 101 to 200 of P protein (Fig. 5C and D).

### The inhibition of IFN-β production by P protein is not dependent on degradation or phosphorylation of IRF3.

**FIG 6:**
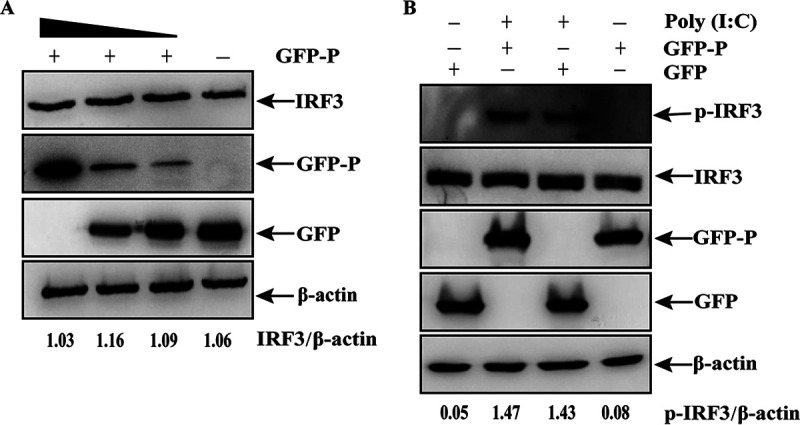
The effect of P protein on degradation or phosphorylation of IRF3. (A) HEK-293T cells transfected with different doses of expression plasmid P (GFP-P) or GFP. At 36 hpt, the cells were processed, and the expression of IRF3, GFP-tag protein, and β-actin was measured using Western blotting. (B) HEK-293T cells were transfected with expression plasmid P (GFP-P) or empty plasmid (GFP). After 24 h, the cells were transfected with poly (I·C) for 12 h and analyzed using Western blotting for p-IRF3, IRF3, GFP-P, GFP, and β-actin.

Virus-mediated degradation of IRF3 inhibits IFN-β production (42). To explore whether IRF3 was degraded by the P protein, HEK-293T cells were transfected with different doses of GFP-P plasmids, and the expression of endogenous IRF3 was measured. IRF3 expression did not change significantly with an increase in P protein levels (Fig. 6A), indicating that the inhibitory role of the P protein was not related to IRF3 degradation. IRF3 phosphorylation is critical for IFN-β production (43), which prompted us to investigate whether IRF3 phosphorylation was affected by P protein in cells treated with poly (I·C). HEK-293T cells overexpressing GFP-P or GFP were transfected with poly (I·C) to analyze the degree of IRF3 phosphorylation. Although IRF3 phosphorylation was markedly enhanced by poly (I·C) treatment in cells transfected with GFP-P or GFP plasmids, the phosphorylation level of IRF3 was not obviously different between GFP-P-expressing cells and GFP-expressing cells (Fig. 6B). These results suggest that aMPV/C P protein inhibited IRF3 activity neither by degrading IRF3 nor by affecting its phosphorylation level.

### aMPV/C P protein blocks IRF3 nuclear translocation.

**FIG 7:**
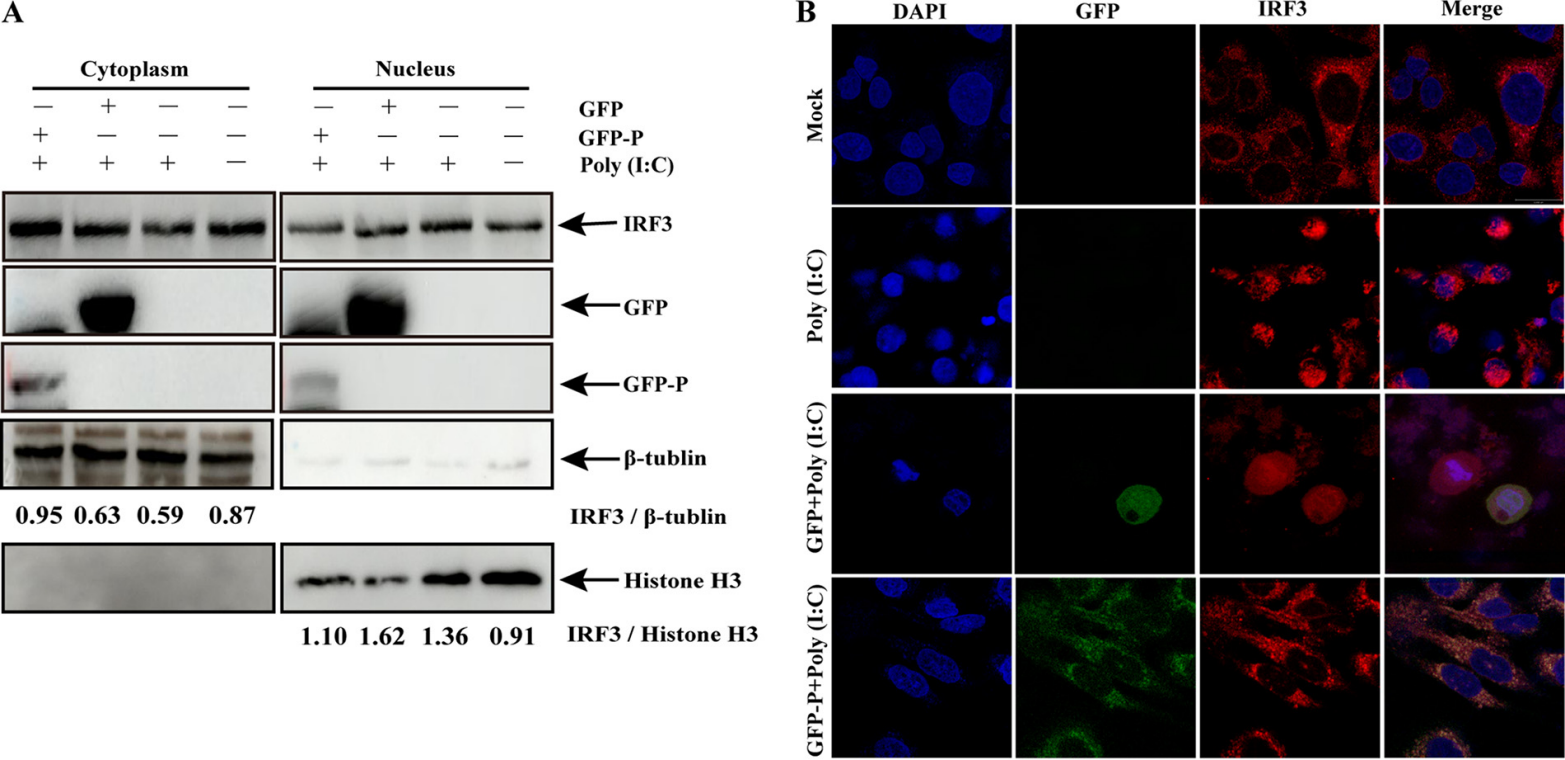
aMPV/C P protein blocks IRF3 nuclear translocation. (A) HEK-293T cells were transfected with expression plasmid P (GFP-P) or empty plasmid (GFP) for 24 h and subsequently transfected with poly (I·C) for 12 h. Cytoplasmic extracts and nuclear extracts were extracted and detected using Western blotting for p-IRF3, IRF3, GFP-P, GFP, and β-actin. (B) A549 cells were transfected and treated with poly (I·C), as described in the legend to panel A. Cells were stained with anti-IRF3 antibody. Cell nuclei were stained with DAPI (blue). Images were obtained using confocal microscopy. Scale bars, 20 μm.

IRF3, a vital inducer of IFN-I production in antiviral immunity, is activated by phosphorylation, following which it translocates into the nucleus to perform its regulatory function (44). On the basis of the results of co-IP between P protein and IRF3, we explored whether inhibition of IFN-I production by the P protein blocks IRF3 entry into the nucleus through their interaction; HEK-293T cells were transfected with GFP-P plasmids and then treated with poly (I·C). The nuclear and cytoplasmic extracts were analyzed for measuring endogenous IRF3 levels. Following poly (I·C) treatment, GFP-P-expressing cells exhibited an increase in endogenous IRF3 in the cytoplasm and a decrease in endogenous IRF3 in the nucleus compared with GFP-expressing cells (Fig. 7A). In addition, nuclear translocation of endogenous IRF3 was examined using confocal microscopy. Endogenous IRF3 was localized mainly to the cytoplasm in mock-transfected A549 cells, whereas poly (I·C) treatment mediated endogenous IRF3 transfer into the nucleus (Fig. 7B). In GFP-P-expressing cells, endogenous IRF3 was restricted from entering the nucleus, and the P protein and IRF3 colocalized in the cytoplasm (Fig. 7B). Collectively, these results indicate that the aMPV/C P protein inhibits the nuclear translocation of IRF3 via their interaction.

## DISCUSSION

The innate immune response is the first line of defense against pathogens, in which IFN-I plays an antiviral role by directly suppressing viral replication or inducing adaptive immunity to clear viruses. Clarifying the mechanisms underlying IFN-I inhibition by viruses will help us develop drugs or vaccines to prevent and control viral infections. Through this study, we demonstrate that the aMPV/C P protein inhibits RIG-I-like receptor (RLR) signaling and subsequently targets IRF3 to downregulate IFN production. Both aMPV/C infection and P protein expression suppressed IFN-β production. Further results showed that the aMPV/C P protein prevents the nuclear transportation of IRF3 by specifically interacting with IRF3 and finally inhibits the regulatory activity of IRF3.

Many viral infections, such as porcine circovirus 2 (PCV2) (45), porcine epidemic diarrhea virus (PEDV) (46), and SVV (22) infections, trigger innate immunity inhibition or evasion, which guarantees viral proliferation. During viral infection, viral proteins manipulate various strategies or target different IFN-associated proteins to inhibit IFN-I production or downstream ISGs and finally facilitate virus proliferation. For example, the African swine fever (ASFV) E120R protein interacts with IRF3 and interferes with the binding of IRF3 to TBK1, which finally suppresses IRF3 phosphorylation and IFN production (47). The influenza A virus (IAV) PB1 protein is associated with a selective autophagic receptor neighbor of BRCA1 (NBR1), which recognizes and degrades ubiquitinated MAVS by autophagy (48). The duck Tembusu virus (DTMUV) NS2A protein strongly blocks stimulation of interferon gene (STING)-induced signal transduction, which subsequently blocks STING–STING binding and TBK1 phosphorylation (49). In addition, viruses of the *Paramyxoviridae* family, such as PPRV, MV, NiV, and NDV, interfere with IFN signal activation (24 to 27). The viral P protein plays an important role in blocking IFN activation in the above viruses, which led us to speculate that the aMPV/C P protein has a similar inhibitory effect. The inhibitory effect of IFN-β was first measured in aMPV/C-infected DF-1 cells, which showed that aMPV/C infection reduced IFN-β production and ISG gene expression (Fig. 1). Additionally, we confirmed that P protein was responsible for the failure of IFN-β production (Fig. 1), which indicated that the aMPV/C P protein performed a similar function as the P protein of other *Paramyxoviridae* viruses.

hMPV has a close evolutionary relationship with aMPV/C (11), and hMPV-B1 P protein has been reported to prevent RIG-I-mediated sensing of viral RNA and inhibit IFN-I activation (29), which suggests that the inhibitory effect of the aMPV/C P protein may focus on RLR signaling. The lack of RIG-I protein in chicken cells indicates that MDA5 acts as a complementary ligand in the recognition of RNA (33). Therefore, we analyzed the inhibitory targets of P protein in the RLR pathway in DF-1 cells and found that the P protein was responsible for the inhibition of IRF7 activity (Fig. 2), which was different from the inhibitory mechanism of the hMPV-B1 P protein, indicating that the aMPV/C P protein has a novel function in regulating IFN-β production.

IRF7 is a crucial regulator of IFN-I against viral infections and is activated by the signaling cascade response from recognizing pathogenic nucleic acids (50). During viral infections, several viruses utilize various strategies to block IRF7 activity; these inhibition mechanisms may be based on the interaction between viral proteins and IRF7. For example, the 1 to 55 region of PEDV M protein disrupts IRF7 function and exerts antagonistic effects on IFN-I production by interacting with IRF7 (46). The EBV LF2 tegument protein specifically interacts with the CAD domain of IRF7 and subsequently inhibits the dimerization of IRF7 and IFN-α production (38). These reports prompted us to investigate whether the aMPV/C P protein interacts with IRF7. Our results confirmed this hypothesis and demonstrated that the activation domain (AD) of IRF7 is a key interaction domain that interacts with the P protein (Fig. 3).

IRF7 plays a particularly critical role in regulating the IFN pathway because of the lack of IRF3 in chicken cells or other avian cells (38 to 40), and its function is similar to that of IRF3 in mammalian cells (41), indicating that the aMPV/C P protein may antagonize IRF3 activity. HEP-2 cells, a human cell line, can be infected by aMPV, which provides a premise for studying the effect of the P protein on IRF3 activity in mammalian cells (51). Our results showed that the aMPV/C P protein was responsible for the antagonistic effect on IRF3 in HEK-293T cells and interacts with IRF3 (Fig. 4), and the critical interaction domain (101 to 200 aa) in P protein was identified (Fig. 5). The lack of commercial chicken antibodies against IRF7 and phosphorylated IRF7 prompted us to deeply explore the inhibitory mechanism of the P protein against IRF3.

Viral proteins targeting IRF3 can antagonize IRF3 activity through various strategies, such as proteasomal degradation of IRF3 (52), inhibition of IRF3 phosphorylation (25), and disturbance of IRF3 nuclear translocation (53). In our study, we found no differences in IRF3 expression with the increase of the P protein expression, suggesting that IRF3 was not degraded by the P protein (Fig. 6A). The inactive IRF3 localized in the cytoplasm is phosphorylated and dimerized after stimulation by viral infection, which allows for its nuclear translocation and subsequent induction of IFN genes (54, 55). IRF3 dimerization mainly depends on IRF3 phosphorylation (56 to 58); therefore, the levels of phosphorylated IRF3 were evaluated in cells expressing the P protein. Our results showed no significant differences in the level of IRF3 phosphorylation between GFP-P-expressing cells and GFP-expressing cells (Fig. 6B), suggesting that the inhibition of IFN-β production by the P protein was not achieved by the inhibition of IRF3 phosphorylation. IRF3 is phosphorylated by TBK1 and then transferred into the nucleus, where it binds and regulates the IFN-β promoter (59). Moreover, the proteins of some viruses can disrupt the association between TBK1 and IRF3, blocking IRF3 phosphorylation (24, 25). The observation that IRF3 phosphorylation was not affected by aMPV/C P protein suggests that the expression of the P protein could not disrupt the interaction between TBK1 and IRF3 and further affect dimerization of IRF3. Interestingly, nuclear translocation of IRF3 was inhibited by the P protein (Fig. 7), and many viral proteins also exhibit this inhibitory effect (23, 24, 53). However, in contrast to the inhibition mechanism of these viral proteins, including that of other P proteins in *Paramyxoviridae*, the aMPV/C P protein reduced IFN-I production only by inhibiting the nuclear translocation of IRF3. The aMPV/C P protein also shows different inhibitory targets and mechanism of antagonizing IFN response from P protein of hMPV (29). These data indicated that aMPV/C P protein has a special inhibitory mechanism unlike P proteins of other *Paramyxoviridae* viruses. Interestingly, this inhibitory effect of the aMPV/C P protein was similar to that of the SARS-CoV-2 NSP5 protein (60). The failure of SARS-CoV-2 NSP5 protein to elicit IFN-I production was dependent on specifically suppressing the nuclear translocation of IRF3, but not on cleaving IRF3 or impairing IRF3 phosphorylation. NSP5 proteases have different roles in the suppression of IFN-I production in human coronaviruses and animal coronaviruses. For instance, the feline infectious peritonitis virus is an alphacoronavirus, and its viral NSP5 protein inhibits IFN response through cleaving IKK-γ (61). NSP5 of porcine deltacoronavirus cleaves STAT2 and DCP1A to antagonize IFN production (62, 63). These reports suggest that even among viruses of the same family, similar viral proteins have their own unique functions. However, the precise mechanism of IFN production antagonized by the aMPV/C P protein requires further investigation in the future.

In summary, our data revealed a novel antagonistic mechanism by which aMPV/C P protein blocks RLR signaling and IFN-I production. We confirmed that the aMPV/C P protein blocked IRF3 nuclear translocation. These findings suggest a new strategy by which aMPV/C escapes the host immune response by P protein, and provide new insights into the pathogenesis of aMPV/C.

## MATERIALS AND METHODS

### Cells, virus culture, antibodies, and plasmids construction.

DF-1, HEK-293T, and A549 cells were originally obtained from the American Type Culture Collection (ATCC) and cultured in Dulbecco’s modified Eagle’s medium (DMEM; Gibco, NY, USA) containing 10% fetal bovine serum (FBS) (Gibco, Life Technologies, USA), streptomycin, and penicillin at 37°C in a 5% CO_2_ incubator.

aMPV/C strain JC was isolated from meat-type chickens with respiratory diseases as described previously (11), and propagated in Vero cells. The virus titer measured was 10^5.5^ of the 50% tissue culture infectious dose (TCID_50_) per mL, and the JC strain was stored at −80°C.

The commercial antibodies used in this study were as follows: rabbit anti-Flag antibody and rabbit anti-GFP antibody (HuaAn, China), rabbit anti-IRF3 antibody and rabbit anti-histone H3 antibody (ABclonal, China), rabbit anti-p-IRF3 antibody (CST, USA), rabbit anti-β-tubulin antibody (MBL, Japan), rabbit anti-HA antibody, mouse anti-β-actin antibody, horseradish peroxidase (HRP)-conjugated anti-rabbit secondary antibody and HRP-conjugated anti-mouse secondary antibody (Sangon Biotech, China), and tetramethyl rhodamine isothiocyanate (TRITC)-conjugated goat anti-rabbit secondary antibody (Sigma-Aldrich, USA). The mouse antiviral N or P protein antibody was prepared in our laboratory.

The full-length P gene and various truncated P genes (1 to 300, 301 to 600, 601 to 885, 1 to 600, and 301 to 885 nucleotides [nt]) amplified from the cDNA of aMPV/C was cloned into the pCMV-GFP-C1 or pCMV-HA vector. The full-length IRF7 gene, various truncated IRF7 genes (1 to 429, 1 to 909, 430 to 909, 427 to 1476, and 907 to 1476 nt), and the innate immune genes encoding MDA5, MAVS, and TBK1 from DF-1 cells were inserted into the pCMV-Flag N vector. IRF3 was obtained from HEK-293T cells and cloned into the pCMV-Flag-N vector. All the constructed plasmids were verified by DNA sequencing, and all primers used for plasmid construction are listed in Table 1. The chicken or human firefly luciferase reporter plasmids (pIFN-β-luc, pIRF7-luc, pNF-κB-luc, and pIRF3-luc) and Renilla luciferase reporter plasmid pRL-TK used in this study were kindly provided by Zhenhai Chen (53) and Kai Li (53, 64).

**Table 1:** Primers and corresponding sequences

Primers	Sequence (5′-3′)
qchicken-IFN-βF	CCTCAACCAGATCCAGCATT
qchicken-IFN-βR	GGATGAGGCTGTGAGAGGAG
qchicken-GADPHF	TGATGCCCCCATGTTTGTGA
qchicken-GADPHR	GATGGCATGGACAGTGGTCA
qchicken-MX1F	GTTTCGGACATGGGGAGTAA
qchicken-MX1R	GCATACGATTTCTTCAACTTTGG
qchicken-OASLF	GGAGTCAGCATCACCAGTCC
qchicken-OASLR	GTCGTAAGCAGGCAGGATGT
qhuman-IRF3F	GTGGGAGTTCGAGGTGACAG
qhuman-IRF3R	ACGTAGCTCATCACTCCCCT
qhuman-GADPHF	TCCTGCACCACCAACTGTTT
qhuman-GADPHR	GGATGATGTTCTGGTGGGCA
chicken-MDA5F	GGAAGCTTATGTCGGAGGAGTGCCGAGACGAGC
chicken-MDA5R	AAGTCGACAGTTAATCTTCATCACTTGAAGGACAA
chicken-MAVSF	CCGAATTCCCATGGGTTTCGCCGAGGACAAAGTGT
chicken-MAVSR	CCGTCGACAACTATTTCTGCAATCGTGTGTACACC
chicken-TBK1F	CCAAGCTTATGCAGAGCACCTCGAATTACCTCT
chicken-TBK1R	CCGTCGACAGTTAGATACAGTCCACATTCCTCAGG
chicken-IRF7F	TTAAGCTTATGGCAGCACTGGACAGCGAGGGGG
chicken-IRF7R	CCGTCGACCCTCAGTCTGTCTGCATGTGGTATTGC
IRF7DBDF	TTAAGCTTATGGCAGCACTGGACAGCGAGGGGGACGCCC
IRF7DBDR	AAGGTACCTCACGTCAGGGCTGCTGCAGCAAC
IRF7ΔDBDF	CCAAGCTTATGCAGGTGGATTTGGACCTGCTG
IRF7ΔDBDR	CCGGTACCTCAGTCTGTCTGCATGTGGTATTG
IRF7ΔIRDF	TTAAGCTTATGGCAGCACTGGACAGCGAGGGGGACGCCC
IRF7ΔIRDR	AAGGTACCTCAGTCCACCTGCTCCTGGTAGACC
IRF7IRDF	TTAAGCTTATGGACAGCCGCTGTGTGCTGGCCTA
IRF7IRDR	CCGGTACCTCAGTCTGTCTGCATGTGGTATTG
IRF7ADF	CCAAGCTTATGCAGGTGGATTTGGACCTGCTG
IRF7ADR	AAGGTACCTCAGTCCACCTGCTCCTGGTAGACC
HA-PF	CCGGATCCCCATGTCCTTTCCTGAGGGGAAAGATA
HA-PR	GCGAATCCCTACATAGTAAGAGAGTATAGGTCA
human-IRF3F	CCAAGCTTATGGGAACCCCAAAGCCACGGATCC
human-IRF3R	AAGGTACCATTGGTTGAGGTGGTGGGGAACAGGG
GFP-PF	CCGAATTCGATGTCCTTTCCTGAGGGGAAAGATA
GFP-PR	CCGTCGAC CTACATAGTAAGAGAGTATAGGTCA
GFP-PF (1 to 100 aa)	CCGTCGACATGTCCTTTCCTGAGGGGAAAGATA
GFP-PR (1 to 100 aa)	AAGGATCCTTACTCCTCGTACAACCGGCTTGCATCC
GFP-PF (101 to 200 aa)	CCGTCGACATGGTCTTTGCTCCTACGAGTGATG
GFP-PR (101 to 200 aa)	AAGGATCCTTAGGGCCCAGCTGTGGCAACATTG
GFP-PF (201 to 295 aa)	AAGTCGACATGACTGCAGCTAGAGACGGCATCC
GFP-PR (201 to 295 aa)	CCGGATCCCTACATAGTAAGAGAGTATAGGTCA
GFP-PF (1 to 200 aa)	CCGTCGACATGTCCTTTCCTGAGGGGAAAGATA
GFP-PR (1 to 200 aa)	AAGGATCCTTAGGGCCCAGCTGTGGCAACATTG
GFP-PF (101 to 295 aa)	CCGTCGACATGGTCTTTGCTCCTACGAGTGATG
GFP-PR (101 to 295 aa)	CCGGATCCCTACATAGTAAGAGAGTATAGGTCA

### Luciferase assay.

DF-1 or HEK-293T cells cultured in 24-well plates were cotransfected with pIFN-β-luc, pIRF7-luc, pNF-κB-luc, or pIRF3-luc and pRL-TK, together with HA-P and expression plasmids MDA5, MAVS, or TBK1 using Lipofectamine 2000 (Invitrogen, USA), according to the manufacturer’s instructions. At 24 h posttransfection (hpt), cells were transfected with or without poly (I·C) (1 μg/well) (Invitrogen, USA) for 12 h and subsequently processed and analyzed using a dual-luciferase reporter assay system (Beyotime, China). The results from three independent experiments are presented as the relative luciferase activity normalized to Renilla luciferase activity.

### Real-time quantitative RT-PCR (qPCR).

Total RNA from the cells was extracted with TRIzol reagent (Invitrogen, USA), and the extracted RNA was reverse transcribed using HiScript III RT SuperMix (Vazyme, China) to generate cDNAs. The synthesized cDNA was determined by RT-qPCR using *Taq* Pro Universal SYBR qPCR Master Mix (Vazyme, China) and measured with a LightCycler 96 system (Roche, Switzerland). The relative mRNA levels (chicken-IFN-β, chicken-MX1, chicken-OASL, human-IFN-β, or human-IRF3) were normalized to chicken or human GAPDH mRNA levels. The relative fold differences were calculated using the 2–ΔΔCT method, and all samples were examined in triplicate on the same plate. All RT-qPCR primers used in this study are listed in Table 1.

### Coimmunoprecipitation and Western blotting.

HEK-293T cells were cotransfected with full-length pCMV-Flag-IRF7, truncated pCMV-Flag-IRF7, pCMV-Flag-IRF3 and pEGFP-P, truncated pEGFP-P, or pEGFP-C1 for 36 h, or DF-1 cells transfected with pCMV-Flag-IRF7 were infected with aMPV/C for 72 h. The cells were lysed with NP40 lysis buffer containing PMSF (Beyotime, China), and the supernatant from the centrifuged cells was immunoprecipitated with anti-GFP agarose (Lablead, China) or anti-Flag agarose (Sigma, USA) at 4°C on a roller. The agaroses were washed five times, and the immunoprecipitates were harvested and analyzed by Western blotting. For Western blotting, cell lysates were quantified, subjected to standard sodium dodecyl sulfate-polyacrylamide gel electrophoresis (SDS-PAGE), and transferred onto a nitrocellulose transfer membrane (Pall, NY). The membranes were blocked with 5% nonfat milk, incubated with the appropriate primary and secondary antibodies, and visualized using an AMERSHAM ImageQuant800 chemiluminescence imaging system (GE, USA).

### Confocal imaging.

DF-1, HEK-293T, and A549 cells cultured in Nubc glass-bottom dishes were transfected with the appropriate plasmids for 24 or 36 h. For confocal imaging, cells were fixed with 4% paraformaldehyde (PFA) and subsequently blocked with 5% nonfat milk, followed by incubation with primary antibodies and secondary antibodies (secondary tetramethylrhodamine-6-isothiocyanate [TRITC]-conjugated anti-rabbit antibody). The nuclei were stained with 4′,6-diamidino-2-phenylindole (DAPI) (Sigma-Aldrich, USA) and observed using a confocal microscope system (model LSM880; Zeiss, Germany).

### Nuclear/cytoplasmic separation.

The cells were lysed, and the cytoplasmic and nuclear proteins were extracted using a nuclear/cytosol fractionation kit (Beyotime, China), according to the manufacturer’s protocols. The separation purity of the nuclear and cytoplasmic proteins was assessed using Western blotting with anti-histone H3 and anti-β-tubulin antibodies, respectively.

### Statistical analysis.

Data are presented as the means ± standard deviations (SD). Differences were determined by one-way analysis of variance or Student's *t* test using the GraphPad Prism 9.0 software (GraphPad Software, CA, USA). For all experiments, *P* values < 0.05 were considered statistically significant.
